# Gut Microbiota and Acute Myeloid Leukemia: State of the Art, Clinical Signals, and Translational Opportunities

**DOI:** 10.3390/antibiotics15040417

**Published:** 2026-04-20

**Authors:** Maria Eugenia Alvaro, Santino Caserta, Enrica Antonia Martino, Mamdouh Skafi, Antonella Bruzzese, Nicola Amodio, Eugenio Lucia, Virginia Olivito, Caterina Labanca, Francesco Mendicino, Ernesto Vigna, Fortunato Morabito, Massimo Gentile

**Affiliations:** 1 Hematology Unit, Department of Onco-Hematology, Azienda Ospedaliera of Cosenza, 87100 Cosenza, Italy; m.eugenia983@gmail.com (M.E.A.); s.caserta@aocs.it (S.C.); enricaantoniamartino@gmail.com (E.A.M.); a.bruzzese@aocs.it (A.B.); e.lucia@aocs.it (E.L.); v.olivito@aocs.it (V.O.); c.labanca@aocs.it (C.L.); frmendicino@gmail.com (F.M.); ernesto.vigna@aocs.it (E.V.); 2 Emergency and Internal Medicine Department, Saint Joseph Hospital, Jerusalem 91192, Palestine; mamdouh272844@gmail.com; 3 Department of Experimental and Clinical Medicine, University of Catanzaro, 88100 Catanzaro, Italy; amodio@unicz.it; 4 AIL Sezione di Cosenza, 87100 Cosenza, Italy; f.morabito53@gmail.com; 5 Department of Pharmacy, Health and Nutritional Science, University of Calabria, 87036 Rende, Italy

**Keywords:** acute myeloid leukemia, gut microbiota, dysbiosis, short-chain fatty acids, fecal microbiota transplantation, induction chemotherapy, hematologic recovery

## Abstract

Acute myeloid leukemia (AML) remains a highly morbid malignancy in which outcomes are constrained not only by disease refractoriness and relapse, but also by therapy-related toxicity—particularly infections, mucosal injury, and delayed hematopoietic reconstitution. The gut microbiota has emerged as a potentially modifiable layer of host vulnerability and resilience during AML treatment. Microbiome disruption is detectable already at diagnosis, even in antibiotic-naïve patients, and is often characterized by reduced community diversity, depletion of anaerobic taxa linked to short-chain fatty acids (SCFAs) production, and enrichment of pathobiont-associated profiles. During induction, cytotoxic therapy and antimicrobials precipitates diversity loss, domination events, and persistent shifts beyond discharge. Clinically, the most consistent translational signal is the association between baseline or early-treatment microbiome features and infectious outcomes, while emerging data suggest that diagnosis-time microbiome structure may also relate to hematologic recovery kinetics. Mechanistic models converge on pathways linking barrier integrity, microbial metabolites (notably butyrate and other SCFAs), immune calibration, and inflammatory translocation of microbial products. These insights support hypotheses: antimicrobial stewardship may preserve microbiome function; ecosystem repair strategies such as autologous fecal microbiota transfer (A-FMT) are feasible and can restore community structure; and metabolite or nutritional interventions merit evaluation in immunocompromised hosts. Regimen-specific microbiome effects and microbiome–drug interactions suggest that treatment choice could have downstream microbiome-mediated consequences. We synthesize evidence, outline interventional concepts, and define methodological priorities for next-generation trials assessing causality and clinical benefit. Progress will require longitudinal sampling, multi-omic integration (metabolomics, resistomics, and barrier/inflammatory biomarkers), and interventional designs linking microbiome dynamics to clinically meaningful outcomes.

## 1. Introduction

The gut microbiota refers to the diverse community of microorganisms inhabiting the gastrointestinal tract—predominantly the colon—including bacteria, viruses, fungi, and archaea, whose collective activity contributes to host physiology through metabolic functions, mucosal immune education, and maintenance of intestinal integrity [1]. Throughout this review, “microbiota” refers to the microorganisms themselves, whereas “microbiome” (or “gut microbiome”) denotes their collective genetic content and functional potential; many human studies use “microbiome” as shorthand for community composition and function inferred by sequencing-based profiling.

In physiological conditions, the intestinal ecosystem is typically dominated by the phyla Firmicutes and Bacteroidota, with variable contributions from Actinobacteriota, Proteobacteria, and Verrucomicrobiota. A central functional output of this ecosystem is the generation of short-chain fatty acids (SCFAs)—notably acetate and butyrate—whose producers include taxa such as *Faecalibacterium* and *Roseburia* (butyrate-associated) and several acetate-linked genera (e.g., *Akkermansia*, *Bacteroides*, *Bifidobacterium*, *Prevotella*, *Ruminococcus*, and *Streptococcus*) [2,3]. SCFAs contribute to epithelial barrier integrity, immunomodulation, and colonization resistance, limiting pathogen expansion [4].

Dysbiosis denotes a sustained disruption of this ecological balance, commonly characterized by reduced microbial diversity, depletion of functionally beneficial anaerobic commensals, and increased relative abundance—or frank “domination”—by taxa that can behave as pathobionts, particularly under conditions of mucosal injury and antimicrobial pressure [5].

In hematologic settings, dysbiosis is clinically consequential because barrier dysfunction, inflammatory amplification, and loss of colonization resistance may converge to increase vulnerability to infections and other treatment-related complications [6].

In AML, gut microbiota alterations are evident at diagnosis—even in antibiotic-free patients—and are then amplified during intensive chemotherapy and subsequent treatment phases. These changes include reduced alpha diversity, loss of SCFA-producing commensals, enrichment of oral and pathobiont-associated bacteria, and persistent community shifts that may not revert to pre-treatment states [7]. Such alterations have been linked not only to infectious outcomes but also to cachexia-like phenotypes and hematologic recovery metrics, suggesting that the microbiome may capture dimensions of host resilience not reflected by leukemic genomics alone [8].

Acute myeloid leukemia (AML) is an aggressive and heterogeneous myeloid malignancy driven by clonal alterations in haematopoietic stem and progenitor compartments, culminating in impaired differentiation, expansion of blasts, and progressive suppression of normal haematopoiesis. Clinically, AML management remains constrained by both disease resistance and therapy-related toxicity [9]. Even with molecularly informed care and targeted agents, long-term outcomes remain suboptimal, particularly in older adults, reflecting a narrower therapeutic window and heightened vulnerability to complications. Risk stratification and treatment allocation continue to rely predominantly on cytogenetic and mutational features. Yet, outcome prediction remains imperfect in a substantial fraction of patients, supporting the need for complementary biomarkers that capture host biology and treatment tolerance [10].

In this context, the gut microbiota has emerged as a biologically plausible and potentially modifiable factor that may influence host vulnerability and resilience during AML therapy, complementing molecular and cytogenetic prognostic frameworks [11].

Microbiome perturbation typically intensifies during induction therapy, a phase characterized by mucosal injury, prolonged hospitalization, and repeated antibiotic exposure [12]. These treatment-related shifts have been linked to barrier dysfunction, infection risk, and cachexia-like phenotypes [8].

Together, these observations position the microbiome as a cross-phase correlate and candidate modifier of infectious risk, mucosal integrity, and recovery trajectories across the AML treatment continuum, from diagnosis through induction and, for many patients, allogeneic transplantation [13].

The goal of this review is to (i) summarize gut microbiota alterations at AML diagnosis and throughout therapy; (ii) synthesize mechanistic links between dysbiosis, barrier dysfunction, immune perturbation, infectious complications, haematopoietic recovery, and potentially leukemic biology; (iii) discuss microbiome–drug interactions; and (iv) outline microbiome-targeted strategies and methodological priorities to enable rigorous interventional studies.

### Literature Search Strategy

This narrative review was informed by a targeted literature search in PubMed, using combinations of MeSH terms and keywords including “acute myeloid leukemia/AML”, “gut microbiota/microbiome”, “dysbiosis”, “induction chemotherapy”, “neutropenia”, “infection”, “bloodstream infection”, “short-chain fatty acids/butyrate”, “intestinal barrier”, “fecal microbiota transplantation/autologous FMT”, and “allogeneic transplantation/graft-versus-host disease”.

We considered publications from 2020 to 2025 and prioritized human studies addressing microbiome features at diagnosis or during therapy, longitudinal designs, clinically annotated cohorts (especially infectious outcomes and recovery kinetics), and interventional or mechanistic studies relevant to AML. Reference lists of key articles were also screened to identify additional eligible reports. Given substantial heterogeneity in patient populations, antimicrobial practices, sampling schedules, sequencing platforms (16S vs. shotgun), and endpoints, we did not perform a formal systematic review or meta-analysis; instead, we synthesized convergent clinical signals and mechanistic hypotheses and highlight methodological priorities for future trials.

## 2. Microbiota Alterations at AML Diagnosis: Dysbiosis Before Therapy

An increasingly important notion in AML microbiome research is that gut ecosystem disruption can be present before any exposure to intensive chemotherapy, rather than being solely a treatment consequence. Cross-sectional studies in antibiotic-free patients at diagnosis consistently demonstrated that AML is associated with a distinct fecal microbial community structure, accompanied by functional and metabolic signatures compatible with altered intestinal homeostasis [14,15].

While diagnosis-time differences have been reported in cohorts described as antibiotic-free, interpretation requires caution. Baseline microbiome structure can be shaped by unrecorded outpatient antibiotic exposure, recent healthcare contacts, concomitant medications (e.g., acid suppressants), comorbidities, and dietary restriction/low intake that often accompany systemic illness. Moreover, inflammatory stress, cytopenias, and early mucosal compromise may themselves promote ecological shifts (including enrichment of oral-associated taxa) independent of leukemia-intrinsic biology [2,14]. Accordingly, current data support the concept that microbiome disruption is detectable at presentation, but they do not establish that dysbiosis is leukemia-specific or causative.

In a multicenter, prospective study of 30 antibiotic-free newly diagnosed AML patients, shotgun metagenomics coupled with fecal, blood, and urine metabolomics revealed compositional differences compared with controls and clinically relevant host phenotypes, including muscle weakness and anorexia, alongside biochemical signals of altered gut function and systemic metabolic stress. This study identified enrichment of oral-associated bacteria in stool—a pattern increasingly linked to epithelial stress and immune perturbation—and a roughly threefold reduction in *Eubacterium eligens*, strongly correlated with muscle strength and circulating citrulline, a marker of enterocyte mass and function. In parallel, *Blautia* and *Parabacteroides* were increased and correlated with appetite-related measures, supporting the concept that diagnosis-associated dysbiosis may align with cachexia-like features in AML [8].

Metabolite-focused datasets further reinforce the concept that SCFA-producing networks are perturbed at presentation [16]. Targeted analyses in newly diagnosed AML reported reductions in butyrate-producing genera (including *Faecalibacterium* and *Roseburia*) and decreases in key SCFAs, particularly butyrate and acetate, while *Enterococcus* is markedly enriched, emerging as a discriminatory signal including *E. faecium* in biomarker analyses. Correlation analyses suggest an inverse relationship between *Enterococcus* abundance and SCFA levels, consistent with a functional readout of microbiome injury rather than a purely taxonomic shift [15,17].

Across these datasets, a coherent diagnosis-stage signature emerges: reduced alpha diversity, enrichment of oral and pathobiont taxa (notably *Enterococcus*), depletion of SCFA-producing commensals such as *Faecalibacterium* and *Roseburia*, perturbation of fecal SCFAs, and parallel cachexia-like phenotypes and markers of epithelial compromise [15].

Although these observations remain associative and cannot alone establish causality, they are biologically coherent given the established roles of SCFAs in supporting epithelial barrier integrity and immunoregulatory tone [18].

Taken together, diagnosis-associated dysbiosis in AML carries two practical implications. First, baseline microbiome features may provide host-level information complementing genomic risk profiling, with potential relevance for prognosis and early complication risk [7]. Second, patients may enter induction therapy with pre-existing ecological fragility, which could amplify the impact of mucosal injury and broad-spectrum antibiotics, predisposing to infectious complications and delayed recovery [19].

In summary, AML patients frequently enter induction therapy with a gut microbiome already shifted away from a diverse, SCFA-rich, barrier-supportive configuration, providing an important ecological context for interpreting therapy-induced perturbations.

## 3. Induction Chemotherapy as an Ecological Stress Test: Diversity Loss, Domination, and Persistent Shifts

Induction chemotherapy represents one of the most profound perturbations of the intestinal ecosystem encountered in clinical medicine [20]. In AML, the combined effects of cytotoxic mucosal injury, prolonged hospitalization, dietary restriction, and repeated exposure to broad-spectrum antimicrobials create a selective landscape that rapidly remodels the gut microbiota. Longitudinal studies consistently demonstrate a steep decline in alpha diversity during induction, accompanied by compositional convergence toward low-complexity communities dominated by a limited set of taxa. This ecological configuration is clinically meaningful: reduced diversity and “domination” states impair colonization resistance, facilitating overgrowth of opportunistic organisms and increasing the likelihood of microbial translocation and bloodstream infection during neutropenia [19,21].

In a large longitudinal cohort with serial stool and oral sampling from baseline through neutrophil recovery, baseline microbiome structure provided predictive information for infectious outcomes [19]. Higher baseline stool Shannon diversity was associated with a greater probability of remaining infection-free during neutropenia (HR approximately 0.36), as well as higher baseline relative abundance of Porphyromonadaceae, which conferred a similar protective association [19,22].

Beyond infections, multi-omics profiling indicates that induction-related dysbiosis is accompanied by host physiological alterations consistent with barrier dysfunction and cachexia-like features. In a longitudinal multi-omics study sampling patients before therapy, at the end of standard induction, and at discharge, intensive treatment transiently impaired gut barrier function and induced long-lasting microbiota shifts characterized by a marked loss of diversity. Clinically, decreased appetite, weight loss, and muscle wasting were common, with time-dependent taxonomic changes including increased Lactobacillaceae and *Campylobacter concisus* at the end of induction, and enrichment of *Enterococcus faecium* and *Staphylococcus* at discharge [21,23,24].

A final practical issue is durability. In a 6-month longitudinal analysis of 52 AML patients (410 stool samples), microbiota trajectories frequently failed to return to baseline after discharge despite major reductions in antibiotic pressure, instead evolving toward new communities highly dissimilar from pre-treatment states. This persistent ecological remodeling is clinically relevant because many patients proceed to consolidation and, in selected cases, allogeneic transplantation—phases in which residual microbiome injury may continue to influence infectious risk and treatment tolerance [25].

Overall, induction chemotherapy in AML functions as an “ecological stress test,” rapidly collapsing diversity, promoting domination states, inducing barrier-associated and cachexia-like phenotypes, and often leaving a persistently altered microbiota that may shape downstream consolidation and transplant outcomes.

## 4. Mechanistic Links: Barrier Integrity, Microbial Metabolites, and Inflammatory Translocation

Current mechanistic models connecting dysbiosis to AML-related outcomes converge on three tightly connected domains: epithelial barrier integrity, immune modulation, and microbiota-derived metabolites that influence both local and systemic inflammation [26,27]. In this framework, the gut barrier is the critical interface limiting the passage of microorganisms and microbe-derived products into the circulation; when barrier function is compromised by disease-related factors, chemotherapy-induced mucosal injury, or antibiotic-driven ecosystem collapse, translocation of inflammatory ligands may amplify systemic immune activation and clinical vulnerability [28].

In AML, this conceptual framework provides a mechanistic bridge between the descriptive dysbiosis observed at diagnosis and during induction, and the clinical endpoints of infection, inflammation, and treatment tolerance.

### 4.1. SCFAs and Butyrate as Barrier-Protective Signals

Among microbial metabolites, SCFAs—and particularly butyrate—are frequently positioned as barrier-supportive mediators. Butyrate is a major energy source for colonocytes and maintains epithelial homeostasis by strengthening tight junction architecture and modulating anti-inflammatory signaling. Acetate and propionate, which together with butyrate constitute most luminal and circulating SCFAs, also contribute to epithelial and systemic immunometabolic regulation via receptor-mediated pathways (e.g., FFAR2/FFAR3) and may shape inflammatory tone; however, compared with butyrate, their specific roles in AML remain less well defined and represent an important knowledge gap [26,29].

Preclinical evidence (murine AML models). Experimental work has explored whether microbiota-derived SCFAs may modulate barrier integrity and inflammatory translocation. A preclinical study integrating human AML samples with murine AML models reported reduced gut microbiota diversity and significant decreases in intestinal butyrate. Antibiotic-induced dysbiosis accelerated AML progression in mice, whereas fecal microbiota transplantation (FMT) reversed these detrimental effects. Mechanistically, AML was associated with disruption of epithelial tight-junction programs (e.g., reduced ZO-1 and claudin-1 and increased claudin-2), and butyrate treatment partially restored junctional protein expression, supporting a direct barrier-protective role [26].

Barrier injury increases systemic exposure to microbial inflammatory products. In patients with AML, higher circulating LPS has been reported in association with barrier injury, whereas in murine models experimental manipulation with antibiotics, FMT, or butyrate modified LPS readouts—supporting a testable mechanistic hypothesis: loss of SCFA-producing anaerobes → reduced butyrate → barrier fragility → increased LPS translocation → inflammatory amplification of disease and complications [30].

Clinical datasets at diagnosis are directionally consistent: newly diagnosed AML patients show depletion of putative SCFA-associated genera (e.g., *Faecalibacterium* and *Roseburia*), reduced acetate and butyrate, and enrichment of *Enterococcus* with a strong inverse correlation between *Enterococcus* abundance and SCFA levels [15,26,30].

Overall, preclinical data support a mechanistic hypothesis linking SCFA depletion (particularly butyrate) to barrier vulnerability and increased translocation of microbial products, whereas human studies are currently consistent with association between SCFA-linked dysbiosis and markers compatible with barrier stress.

### 4.2. Colonization Resistance and Domination States

A second mechanistic axis is colonization resistance: the community-level capacity of a diverse microbiota to suppress opportunistic expansion. When diversity collapses, “domination” by a single taxon or limited consortium becomes more likely, reducing ecological buffering and increasing susceptibility to invasive infection and inflammatory complications [31].

Although AML induction is distinct from allogeneic transplantation, both share profound antibiotic exposure and mucosal injury; post-HCT data indicate that low diversity dysbiosis, loss of anaerobic commensals, and *Enterococcus* expansion/domination associate with adverse outcomes, offering a clinically relevant parallel model [21,32].

AML-specific longitudinal multi-omics data also show treatment-associated shifts compatible with domination dynamics, including enrichment of *Enterococcus faecium* and *Staphylococcus* at discharge after intensive chemotherapy, together with markers consistent with transient barrier impairment [21].

Overall, these mechanistic pathways support the view that dysbiosis may represent more than a bystander feature of therapy and could plausibly influence barrier function and vulnerability to downstream complications; however, in clinical AML these relationships remain to be tested in interventional designs.

The remainder of the review interprets clinical associations (e.g., infections, hematologic recovery) in light of these mechanistic axes—SCFA depletion, barrier injury, and loss of colonization resistance—rather than as purely descriptive correlations.

The key human studies evaluating gut microbiota alterations in AML are summarized in Table 1.

## 5. Clinical Translation: Infections and Hematologic Recovery as Microbiome-Linked Endpoints

### 5.1. Infectious Risk Stratification

The most consistent clinical association in AML microbiome research to date concerns the relationship between baseline or early-treatment gut ecology and subsequent infectious complications during and after induction [36].

In a longitudinal cohort of 97 AML patients undergoing intensive induction chemotherapy with serial oral and stool sampling through neutrophil recovery, higher baseline stool Shannon diversity was associated with a greater probability of remaining infection-free during neutropenia (HR ≈ 0.36). Similarly, a higher baseline relative abundance of Porphyromonadaceae conferred a protective association. Importantly, this work also provides a framework for microbiome-based risk modeling that is directly relevant to supportive care: a Shannon diversity threshold was proposed as an exploratory approach to identify patients at higher risk for infectious complications [19]. However, such models should be viewed as hypothesis-generating and require external validation and harmonized sampling/analytic pipelines before any consideration for routine supportive-care implementation.

Antibiotic exposure emerged not merely as a confounder, but as a major determinant of microbiome injury with downstream clinical implications [37]. Greater antibiotic burden correlated with reduced diversity at neutrophil recovery, and carbapenem exposure > 72 h was associated with lower alpha diversity at recovery and a higher subsequent infection risk [19]. These observations support a translational hypothesis: antimicrobial spectrum and duration shape microbiome trajectories in ways that extend beyond the neutropenic window, potentially influencing later infectious susceptibility and host recovery. While causality cannot be inferred from observational data alone, these findings provide a rational basis to embed microbiome endpoints—such as diversity preservation, prevention of domination, and resistome dynamics—into stewardship-focused interventional studies in AML [38].

Clinically, baseline stool diversity and specific taxa could serve as research-grade biomarkers to stratify infectious risk and to prioritize patients for microbiome-preserving supportive care strategies, rather than as stand-alone tools for routine clinical decision-making.

### 5.2. Hematologic Recovery

Beyond infections, preliminary evidence suggests that the gut microbiome at diagnosis may relate to hematologic recovery kinetics after induction [39]. However, available data derive from limited cohorts and remain susceptible to confounding by supportive-care exposures.

In a profiling study of 27 newly diagnosed adult AML patients, microbiome features at presentation correlated with recovery metrics for platelets, lymphocytes, and neutrophils. Patients with more favorable recovery exhibited higher alpha diversity and enrichment of taxa linked to gut health and fermentative capacity, including *Faecalibacterium*, *Ruminococcus*, *Blautia*, and *Butyricimonas*. These findings support the concept that baseline microbiome ecology may capture aspects of host-level resilience not fully reflected by leukemia genomics alone [33].

Together, the infection and recovery signals justify prospective validation using standardized clinical endpoints, including time to ANC and platelet recovery, transfusion requirements, infection definitions, and inflammatory/barrier biomarkers, with analytic frameworks that treat antibiotic exposure as a time-varying determinant rather than a binary covariate [40].

These observations should therefore be interpreted as exploratory, and future prospective studies should predefine recovery endpoints and model antibiotics as time-varying exposures to clarify whether microbiome features provide independent predictive information.

**Figure 1:**
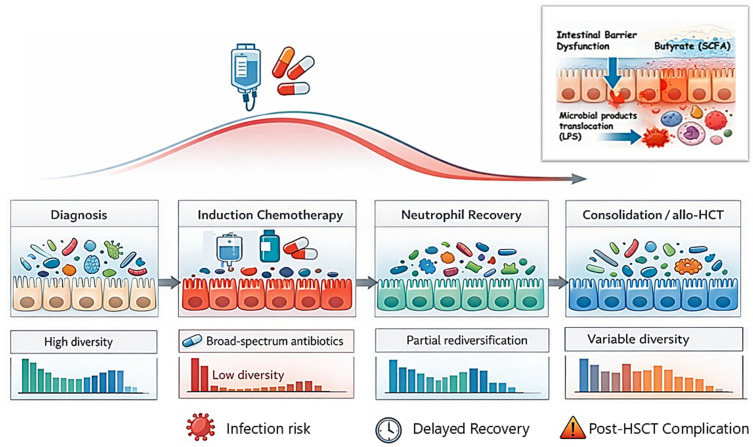
Microbiome–Barrier–Immune Axis Across the AML Treatment Trajectory. Conceptual schematic overwiew of four key phases (diagnosis, induction chemotherapy, neutrophil recovery, consolidation/allo-HCT) that juxtaposes observed microbiome dynamics reported in human AML cohorts with mechanistic hypotheses informed largely by preclinical and related hematologic settings. The upper overlay depicts a representative diversity trajectory (sharp decline during induction with partial recovery thereafter) and phase-specific Shannon diversity bars. The inset illustrates a proposed SCFA (butyrate)–barrier axis and inflammatory translocation (e.g., LPS) as a mechanistic model; it is not intended to imply established causality in clinical AML. Lower panels list clinically relevant endpoints (infections, recovery kinetics, post-transplant inflammatory complications) that have been associated with microbiome features in the literature. Abbreviations: SCFA, short-chain fatty acid; LPS, lipopolysaccharide; allo-HCT, allogeneic hematopoietic cell transplantation.

An integrated overview of microbiome dynamics, barrier disruption, inflammatory translocation, and downstream clinical endpoints across the AML treatment trajectory is provided in Figure 1.

## 6. Microbiome-Targeted Interventions: From Stewardship to Ecosystem Repair

### 6.1. Antibiotic Stewardship as a Microbiome Intervention

Broad-spectrum antibiotics are consistently linked to loss of gut microbial diversity during AML induction, making stewardship a pragmatic, strategy amenable to prospective evaluation with microbiome endpoints, without implying proven microbiome-mediated clinical benefit [41].

In longitudinal AML induction cohorts, greater antibiotic exposure correlated with lower stool Shannon diversity at neutrophil recovery, and carbapenem use for >72 h was associated with significantly reduced diversity and a higher post-recovery infection risk [19].

These data support a testable stewardship hypothesis: balancing empiric breadth with timely reassessment and de-escalation when clinically appropriate, while monitoring microbiome injury as a secondary endpoint. Trial designs could embed microbiome endpoints—such as alpha diversity preservation, prevention of domination events, and resistome dynamics—alongside conventional safety outcomes (e.g., sepsis-related mortality, ICU transfer) [42].

Antibiotic stewardship in AML can thus be reframed as a microbiome-targeted intervention, where ecological preservation becomes an explicit goal, and microbiome metrics serve as secondary endpoints in clinical trials.

### 6.2. Fecal Microbiota Transfer and Autologous Approaches

When dysbiosis is deep and persistent, direct ecosystem restoration becomes conceptually attractive [43]. A phase II, multicenter, single-arm study (NCT02928523) evaluated autologous fecal microbiota transfer (AFMT) in 25 AML patients after intensive chemotherapy and broad-spectrum antibiotics. AFMT restored alpha diversity toward pre-treatment levels, improved similarity to baseline community profiles, and demonstrated feasibility and acceptable short- to mid-term safety in this immunocompromised population [34].

Although single-arm studies cannot establish efficacy for clinical outcomes, AFMT provides a clear operational template for controlled trials assessing endpoints such as bloodstream infections, gut colonization by multidrug-resistant organisms, antibiotic utilization, mucositis severity, and readiness for consolidation or transplantation [44].

At present, AFMT in AML should remain an investigational strategy, with encouraging ecological and feasibility data, but without definitive evidence for improvement in hard clinical outcomes; randomized controlled trials are required to clarify net benefit–risk. At present, AFMT should be considered investigational, and its impact on infections, antibiotic utilization, and treatment readiness remains unproven pending randomized comparisons.

### 6.3. Postbiotics and Metabolite Replacement

Mechanistic work positions SCFAs—particularly butyrate—as barrier-supportive signals that may reduce inflammatory translocation [45]. In translational studies integrating human AML observations with murine models, fecal butyrate and microbial diversity were reduced in AML, antibiotic-induced dysbiosis accelerated leukemic progression, and fecal microbiota transplantation reversed this phenotype. Butyrate supplementation (or administration of a butyrate-producing taxon, such as *Faecalibacterium*) improved barrier integrity, reduced circulating LPS, and attenuated AML progression in vivo [26].

These data provide biological plausibility for “postbiotic” approaches (dietary substrates favoring SCFA producers or direct metabolite replacement). However, clinical translation requires attention to dosing, formulation, tolerability during mucositis, and safety in profoundly neutropenic patients [46].

In AML, postbiotics offer a mechanistically attractive but still experimental approach, aiming to recapitulate key microbial functions (e.g., butyrate production) without transferring whole communities, potentially allowing a more standardized and controllable intervention once safety and efficacy are established.

### 6.4. Diet and Supportive Measures

Dietary modulation could support microbiome resilience, yet implementation in AML is constrained by anorexia, nausea, mucositis, and fluctuating intake during hospitalization [47]. Nevertheless, AML cohorts show clinically relevant links between microbiome alterations and cachexia-related phenotypes at diagnosis and during therapy, supporting the rationale for coordinated nutrition–microbiome strategies [21]. Practical research directions include individualized nutritional support with microbiome-aware endpoints, such as dietary adequacy, body composition, inflammatory markers, SCFA profiles, and diversity trajectories, rather than restrictive regimens that could exacerbate intake reduction. Broader reviews highlight diet as a plausible modifier of microbiota composition and treatment tolerance, motivating prospective studies that integrate standardized dietary assessment and intervention into AML supportive care trials [48].

**Figure 2:**
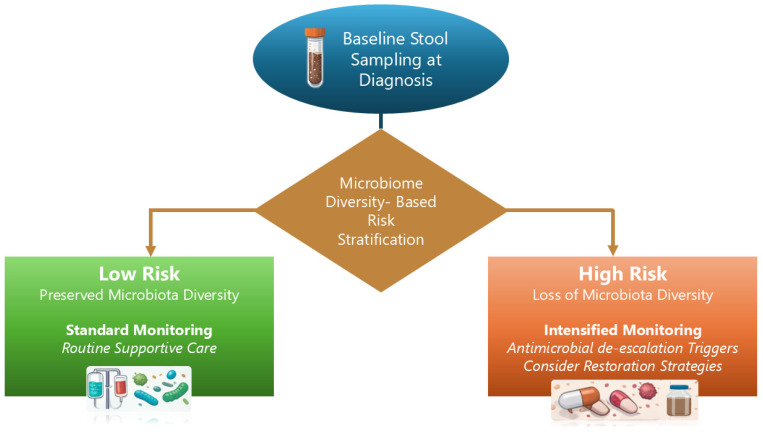
Clinical decision framework for microbiome-informed supportive care. Conceptual flowchart illustrating how baseline stool sampling at diagnosis could be used to derive a microbiome diversity-based risk categorization. Patients with preserved diversity follow standard monitoring and routine supportive care, whereas high-risk trajectories prompt intensified monitoring, predefined antimicrobial de-escalation triggers, and consideration of microbiome restoration strategies within research protocols. This framework is intended to support trial design and hypothesis testing rather than to define a standard-of-care pathway. It is not intended for routine clinical risk stratification outside research settings.

A pragmatic, microbiome-informed supportive-care framework for research/clinical trial contexts is outlined in Figure 2.

Overall, microbiome-targeted strategies in AML span a spectrum from low-risk supportive care optimization (antibiotic stewardship, nutritional support) to experimental ecosystem repair and metabolite-based approaches (AFMT/FMT, postbiotics), which should currently remain confined to clinical trials.

## 7. Microbiome–Drug Interactions and Treatment Response

An emerging theme in AML supportive biology is that antileukemic regimens differ in their collateral effects on the intestinal ecosystem, with potentially downstream consequences for infection risk, inflammatory toxicity, and recovery [49].

In translational studies comparing CPX-351 with conventional “7 + 3,” CPX-351 was associated with preservation of mucosal barrier function and reduced intestinal morbidity, whereas “7 + 3” promoted epithelial damage, inflammation, and increased permeability. Mechanistically, CPX-351 activated a protective host program centered on aryl hydrocarbon receptor (AhR) signaling, inducing IL-22 and reinforcing IL-10–linked regulatory pathways, consistent with improved barrier homeostasis and reduced inflammatory activation [50,51]. A key microbiome-facing implication is that CPX-351 better preserved colonization resistance, with transcriptional signatures suggestive of enhanced epithelial antimicrobial defense and reduced inflammasome-associated signaling compared with “7 + 3.” Functional experiments reinforced this view: fecal microbiota transfer from CPX-351–treated mice mitigated intestinal pathology and reduced endotoxin levels in recipient models, whereas microbiota from “7 + 3”–treated animals transferred a more injurious phenotype [50]. These results derive from experimental models and should be interpreted as hypothesis-generating until corroborated by prospective clinical AML cohorts comparing regimen-specific microbiome and barrier endpoints.

These observations highlight host–microbe crosstalk involving AhR responsiveness to microbial ligands and the production of immunomodulatory metabolites by anaerobes, providing a plausible biological bridge between regimen selection and microbiome trajectories [51].

Collectively, these data suggest that induction regimen choice could exert microbiome-mediated effects extending beyond antileukemic efficacy, potentially influencing the balance between epithelial injury, dysbiosis, and infection susceptibility [19].

A complementary and clinically consequential line of inquiry concerns whether dysbiosis contributes to drug resistance and treatment failure. Convergent mechanisms identified in preclinical and translational studies include: (i) direct drug modification, such as bacterial enzymatic inactivation of anthracyclines; (ii) immune perturbation driven by loss of SCFA-producing taxa and increased inflammatory translocation (LPS-associated “leaky gut”); (iii) metabolite-driven survival pathways, including reduced butyrate-associated HDAC inhibition, acetate-supported metabolic rewiring, and tryptophan–kynurenine/AhR signaling promoting immunoregulatory states [26,38].

While much of this evidence remains mechanistic or associative, it delineates clear research priorities: (i) mapping regimen-specific patterns of microbiome injury in AML patients, (ii) identifying causal taxa and metabolites that modulate chemosensitivity, and (iii) integrating microbiome and metabolomic readouts into trials aimed at reducing resistance and improving durable responses.

At present, these insights should inform hypothesis generation and trial design, rather than guide routine regimen selection, but they underscore the potential of microbiome-informed precision supportive care in AML.

## 8. Methodological Pitfalls and Priorities for the Next Generation of AML Microbiome Trials

Despite rapid advances, AML microbiome research faces predictable—and largely solvable—methodological challenges [40].

The largest source of variability arises from supportive care heterogeneity, particularly antibiotics (choice, timing, duration, prophylaxis strategies, and escalation/de-escalation patterns), which can dominate microbiome trajectories and obscure disease- or regimen-specific signals. Additional inpatient determinants—including antifungals, opioids, parenteral nutrition, diet changes, and the broader “hospital effect”—are variably captured and frequently incompletely reported, increasing residual confounding and limiting cross-study comparability [35,38].

Technical heterogeneity further complicates interpretation. Cohorts differ in sampling density and timing, sequencing approaches (16S amplicon versus shotgun metagenomics), sequencing depth, processing pipelines, and statistical handling of compositional data. Even well-annotated 16S datasets cannot reliably support species- or strain-level inference, motivating the increasing adoption of shotgun approaches, resistomics, and functional profiling for translational claims [38,40].

A practical way forward is the deliberate construction and reuse of multi-omic, longitudinal datasets that integrate microbiota profiles with circulating metabolites, inflammatory/barrier readouts, and granular clinical metadata. For example, a publicly described dataset links 566 stool samples (68 patients) with 260 serum metabolomics (36 patients), with sample-level antibiotic exposure tracked daily. Anchoring events to day 1 of chemotherapy and capturing clinically meaningful landmarks (first neutropenic fever, diarrhea windows, *C. difficile* positivity), enables time-aligned analyses of host–microbe–metabolite dynamics [40]. This resource also highlights limitations to address prospectively in next-generation trials: absence of curated dietary data, incomplete generalizability due to center-specific prophylaxis practices, and the extreme intestinal insults during AML induction.

Translational priorities include harmonized sampling schedules beyond “baseline versus post-treatment” comparisons: baseline pre-antibiotic when feasible; early induction (week 1); onset of neutropenic fever; neutrophil recovery; discharge; pre-consolidation and pre-HCT, given evidence of persistent microbiota shifts and new strain-level stabilization post-hospitalization [40].

Clinical endpoints should be prespecified and uniformly adjudicated including microbiologically documented infection and bloodstream infection, time to ANC/platelet recovery, ICU transfer, and early mortality. Analytic plans should treat antibiotic exposure as time-varying variable rather than binary labels, leveraging day-level data when available [25,33].

Finally, mechanistic bridging should be built into study design: metabolomics to connect taxa to functional outputs, resistomics to capture selection pressure, and barrier/inflammation biomarkers to move beyond descriptive associations toward testable causal hypotheses.

Therefore, a “minimum toolkit” for next-generation AML microbiome trials includes: (i) standardized and densely timed sampling; (ii) detailed, day-level antibiotic and supportive-care data; (iii) at least one additional ’omic layer (e.g., metabolomics or resistomics); (iv) rigorously defined clinical endpoints; and (v) statistical approaches that explicitly model time and treatment exposures.

Microbiome-targeted strategies currently under consideration in AML—ranging from pragmatic microbiome-preserving supportive care to investigational ecosystem-repair and metabolite-based approaches—are summarized in Table 2.

## 9. Conclusions

Accumulating evidence indicates that the gut microbiota is perturbed at AML diagnosis and undergoes a marked, treatment-amplified collapse during intensive induction, with consequences that may extend beyond the neutropenic phase [8,25].

Across observational cohorts, a recurring ecological signature emerges: reduced alpha diversity, depletion of anaerobic commensals linked to SCFA-associated functions, and in some patients an expansion of opportunistic taxa such as *Enterococcus*. These changes are not merely descriptive. Baseline microbiome metrics (including Shannon diversity and specific taxa) correlate with the probability of remaining infection-free during neutropenia, while antibiotic burden—particularly prolonged exposure to broad-spectrum agents such as carbapenems—tracks with deeper diversity loss and higher post-recovery infection risk [15,21].

Emerging data also suggest that microbiome features at diagnosis may relate to recovery kinetics, raising the possibility that the gut ecosystem captures dimensions of host resilience not fully reflected by leukemia genomics alone [33].

Mechanistic studies provide a coherent framework linking dysbiosis to AML-relevant phenotypes: loss of SCFA-producing networks, reduced butyrate, impaired tight-junction integrity, and increased translocation of microbial inflammatory products (e.g., LPS) can amplify systemic inflammation and vulnerability, aligning with human observations of SCFA depletion and barrier-associated markers [26].

From a translational perspective, the most immediate opportunity lies in microbiome-preserving supportive care. Antibiotic stewardship—careful spectrum selection, prompt de-escalation, and minimization of unnecessary duration—represents a pragmatic intervention that can be tested without introducing new biological agents, while incorporating microbiome endpoints such as preservation of diversity, domination events, and resistome contraction [19].

Simultaneously, restoration strategies are moving from concept to feasibility. Autologous fecal microbiota transfer (AFMT) has restored diversity and reconstitute pre-treatment-like community structure in intensively treated AML patients, supporting controlled studies evaluating both microbiome repair and clinically meaningful outcomes [34].

Regimen-specific host–microbiome interactions—exemplified by differential intestinal effects of CPX-351 versus “7 + 3”—underscore that microbiome biology may intersect with therapeutic choice, treatment tolerance, and infection susceptibility [50].

The next phase of the field should therefore prioritize: (i) standardized, longitudinal sampling across key treatment milestones, (ii) multi-omic integration linking taxa to function and host biology, and (iii) interventional designs capable of moving beyond association toward causal inference.

Near-term work should embed microbiome endpoints into stewardship programs and prospective cohorts; medium-term randomized trials should evaluate ecosystem repair (autologous or donor-derived FMT) and postbiotic or nutritional approaches; longer-term goals include microbiome-informed treatment selection and resistance-modifying strategies to improve tolerance and reduce infectious complications in AML.

## Figures and Tables

**Table 1:** Representative studies linking the gut microbiome to clinical outcomes and mechanistic pathways in AML. Summary of key clinical and translational studies evaluating gut microbiota alterations in AML across the treatment course. Studies are grouped by domain (infection risk during induction, hematologic recovery, dysbiosis with barrier/cachexia features, baseline diagnosis-associated dysbiosis, mechanistic preclinical evidence, microbiome restoration, and allo-HCT/GvHD context). For each study, the table reports population/design, profiling approach, principal microbiome findings, and the relevant clinical link. Abbreviations: AML, acute myeloid leukemia; pts, patients; 16S rRNA, 16S ribosomal RNA gene sequencing; ABX, antibiotics; FMT, fecal microbiota transplantation; AFMT, autologous fecal microbiota transplantation; LPS, lipopolysaccharide; allo-HCT, allogeneic hematopoietic cell transplantation; GvHD, graft-versus-host disease.

Domain	Study (Year)	Population/Design	Methods	Main Findings	Clinical Link
Infection risk during induction	Galloway-Peña et al. (2020) [19]	97 AML pts, longitudinal during induction to neutrophil recovery	16S rRNA on stool + oral, serial sampling	Baseline higher Shannon diversity and higher Porphyromonadaceae associated with remaining infection-free; prolonged carbapenem exposure associated with lower diversity at recovery and higher later infections	Association with infection-free course during neutropenia; hypothesis-generating threshold and stewardship signal
Hematologic recovery	Salvestrini et al. (2025) [33]	27 newly diagnosed AML pts, baseline and post-induction	16S rRNA	Higher alpha diversity and enrichment of Faecalibacterium/Ruminococcus/Blautia/Butyricimonas at diagnosis associated with better recovery indicators	Exploratory association with hematologic recovery indices
Dysbiosis + barrier/cachexia during therapy	Pötgens et al. (2023) [21]	AML pts, longitudinal multi-omics across induction/discharge	Microbiome + metabolomics + barrier markers	Loss of diversity, long-lasting compositional change; signals consistent with transient barrier impairment and weight loss	Supportive care implications
Baseline dysbiosis + functional phenotype	Pötgens et al. (2024) [8]	30 antibiotic-free AML at diagnosis vs. controls	Shotgun metagenomics + multi-compartment metabolomics	Microbiome functional shifts; associations with anorexia and muscle weakness; increase in oral bacteria	Links to functional status
Mechanism: butyrate–barrier–LPS	Wang et al. (2022) [26]	AML pts + murine AML models	Microbiome + interventions (ABX, FMT, butyrate)	Antibiotic-induced dysbiosis accelerates AML in mice; FMT reverses; butyrate/Faecalibacterium mitigates barrier damage and LPS leakage	Preclinical mechanistic support (murine; ABX/FMT/butyrate interventions)
Microbiome restoration	Malard et al. (2021) [34]	25 AML pts, phase II single-arm AFMT	Microbiome ecology metrics	AFMT restores alpha diversity and community similarity; feasibility and safety signals	Basis for randomized trials
HCT/GvHD context	van Lier et al. (2023) [35]	Review (allo-HCT)	Synthesis	Post-HCT dysbiosis: low diversity, loss of anaerobes, Enterococcus domination; links to GvHD; microbiome interventions under study	Continuum AML → HCT

**Table 2:** Microbiome-targeted strategies in AML: mechanistic rationale, current evidence, and candidate endpoints for future trials. Approaches are grouped from immediately implementable supportive-care interventions to investigational ecosystem-repair and metabolite-based strategies. Proposed endpoints reflect feasibility in immunocompromised hosts and the need to integrate microbiome and clinical outcomes. Abbreviations: AML, acute myeloid leukemia; SCFA, short-chain fatty acid; FMT, fecal microbiota transplantation; AFMT, autologous fecal microbiota transplantation; MDR, multidrug-resistant; BSI, bloodstream infection; allo-HCT, allogeneic hematopoietic cell transplantation; AhR, aryl hydrocarbon receptor; ICU, intensive care unit; LPS, lipopolysaccharide, IL-, interleukin.

Strategy	Target/Rationale	Current Evidence in AML	Suggested Trial Endpoints	Key Safety/ Implementation Considerations
Antibiotic stewardship (“microbiome-sparing”)	Reduce collateral diversity loss; prevent domination/resistome expansion	Observational induction cohorts link antibiotic burden and carbapenems to diversity loss and later infections	Diversity preservation; domination events; MDR colonization; infection rates; antibiotic days; ICU transfer; early mortality	Must not compromise sepsis management; requires prespecified de-escalation rules and safety monitoring
Autologous fecal microbiota transfer (AFMT)	Ecosystem repair after deep antibiotic/chemo injury	Phase II multicenter single-arm AFMT in AML shows restoration toward baseline ecology and feasibility	α-diversity recovery; community similarity indices; MDR colonization; BSI incidence; mucositis severity; antibiotic utilization; readiness for consolidation/allo-HCT	Donor/autologous sample logistics; timing vs. neutropenia; infection screening; regulatory pathway
FMT/consortia-based restoration (non-autologous)	Rebuild colonization resistance; restore anaerobic networks	Conceptually supported; extrapolations from hematologic settings (incl. HCT literature)	Domination clearance (e.g., Enterococcus); resistome contraction; infection endpoints; inflammatory markers	Higher biosafety/regulatory burden; donor screening; product standardization
Postbiotics/metabolite replacement (e.g., butyrate)	Barrier support; mitigate inflammatory translocation (LPS); immunomodulation	Translational preclinical evidence: butyrate/Faecalibacterium improves barrier metrics and reduces LPS with disease attenuation in vivo	Barrier biomarkers; plasma LPS surrogates; inflammatory panels; SCFA quantification; clinical infection endpoints	Formulation/tolerability during mucositis; dosing; interactions with nutrition; safety in profound neutropenia
Nutrition-informed supportive care (diet quality, substrates for SCFA producers)	Support resilience and recovery of beneficial fermenters	Clinical associations between microbiome and cachexia-like phenotypes at diagnosis/induction; diet reviews in acute leukemia	Dietary adequacy; body composition; SCFA profiles; diversity trajectories; patient-reported outcomes; inflammation	Intake variability (anorexia/nausea); avoids restrictive diets that reduce calories/protein; standardized dietary capture required
Regimen-aware microbiome preservation (e.g., CPX-351 vs. 7 + 3)	Differential mucosal injury and microbiome disruption may influence downstream toxicity	Translational work suggests CPX-351 better preserves barrier/colonization resistance via host–microbe pathways (AhR/IL-22/IL-10)	Barrier function readouts; dysbiosis indices; endotoxin markers; infection rates; recovery kinetics	Confounding by indication; needs controlled clinical validation alongside efficacy endpoints

## Data Availability

Data sharing does not apply to this article, as no new data were generated or analyzed in this study.
